# Constructing and investigating a disulfidptosis-associated LncRNA signature for prognostic prediction in gastric cancer

**DOI:** 10.1007/s12672-026-05209-4

**Published:** 2026-05-20

**Authors:** Namei Li, Zhongyi Tong, Xiaoling She

**Affiliations:** Department of Pathology, The Second Xiangya Hospital, Central South University, Changsha, Hunan 410011 PR China

**Keywords:** Disulfidptosis, Gastric cancer, Prognostic signature, LncRNA

## Abstract

**Background:**

Gastric cancer (GC) prognosis remains poor, necessitating reliable biomarkers. Disulfidptosis represents a promising therapeutic frontier. This study aimed to construct a prognostic model based on disulfidptosis-related LncRNAs (DRLs) and investigate their function.

**Methods:**

DRLs were screened from TCGA-STAD using Pearson correlation and LASSO-COX regression to build a prognostic signature. Its accuracy was evaluated via Kaplan-Meier analysis, time-dependent ROC curves, and decision curve analysis. A key DRL was functionally validated in vitro and in vivo.

**Results:**

We established a six-DRL signature (*AC090809.1*,* CYP4A22-AS1*,* AL356417.2*,* Z94721.2*,* FP325313.3*,* ERICH3-AS1*) that effectively stratified patients into risk groups with distinct survival (*P* < 0.05). The risk score was an independent prognostic factor, with robust predictive AUCs for 1-, 2-, and 3-year survival. AL356417.2 was upregulated in GC and linked to poor outcome. Its knockdown inhibited cell viability, induced F-actin depolymerization, increased cystine uptake, and suppressed tumor growth in mice.

**Conclusion:**

We developed a novel DRL-based prognostic model and identified AL356417.2 as a functional regulator suppressing disulfidptosis, providing mechanistic insights and a potential therapeutic target for GC.

**Supplementary Information:**

The online version contains supplementary material available at 10.1007/s12672-026-05209-4.

## Introduction

The global incidence of gastric cancer (GC) in 2023 reached roughly 1.08 million new cases alongside 770,000 deaths, solidifying its status as the world’s fifth most prevalent cancer, based on the most recent data released by the International Agency for Research on Cancer [1, 2]. China’s 2022 statistics indicate that GC represented 7.43% (ཞ358.7 thousand cases) of all new cancer diagnoses and, more notably, 10.12% (ཞ260.4 thousand deaths) of all cancer deaths. This makes GC the third most lethal cancer in China [3–5]. Despite advancements in diagnosis and treatment, which have raised the 5-year survival rate for early-stage GC in China to over 90%, managing GC remains a formidable challenge, particularly given the persistently poor prognosis for patients with advanced disease [6, 7]. Therefore, further investigation is urgently required to enhance the early detection, prognostic assessment, and therapeutic evaluation of GC, ultimately aimed at improving patient survival.

Regulatory cell death (RCD) is instrumental in disease pathogenesis [8, 9]. Tumor cells, however, often acquire the ability to resist RCD via genetic mutations and other means, a key mechanism by which they drive tumor development and progression [10]. Hence, promoting tumor RCD remains a core therapeutic approach. Adding to known mechanisms like ferroptosis, the team of Professors Boyi Gan and Junjie Chen reported a new form of regulatory cell death in 2023—disulfidptosis. This novel cell death pathway, distinct from apoptosis and ferroptosis, is mechanistically characterized by the abnormal accumulation of intracellular cystine upon glucose deprivation, which leads to NADPH depletion and subsequent disulfide stress. This excessive cystine reduction disrupts redox balance, inducing aberrant intra- or intermolecular disulfide bonds on cysteine residues of actin cytoskeletal proteins, particularly F-actin, ultimately causing cytoskeletal collapse and rapid cell death [11–15]. Current studies propose disulfidptosis-related molecules as potential novel biomarkers for early diagnosis and prognosis in liver cancer [16, 17]. Furthermore, inhibiting disulfidptosis may reduce cell damage in myocardial infarction and stroke [18].

Long non-coding RNAs (LncRNAs), once thought to be non-functional, are now recognized as key regulators in cancer [19]. Their dysregulation contributes to tumor progression by influencing critical processes like cell death, metastasis, and differentiation. Examples include LncRNAs that regulate ferroptosis and cuproptosis [20–22]. Despite this, the specific prognostic value and regulatory mechanisms of disulfidptosis-related LncRNAs (DRLs) in gastric cancer are still not well understood.

Therefore, disulfidptosis holds promise both as a novel treatment for tumors resistant to other therapies and as a source of biomarkers. This study was therefore aimed at building a prognostic model with the goal of elucidating the value of disulfidptosis-related genes in GC.

## Materials and methods

### Analysis of disulfidptosis-related genes

*Data Acquisition* RNA-seq data from 448 GC samples (TCGA-STAD) and a published list of 117 disulfidptosis-related genes (DRGs) were collected [12, 23].

*Gene Screening*: Differential expression analysis of mRNAs and LncRNAs was performed on the TCGA-STAD dataset. The resulting differentially expressed genes were subsequently overlapped with the known DRGs using a Venn diagram, which identified 20 candidate DRGs. DRLs were identified via Pearson correlation analysis in the TCGA-STAD cohort (screening criteria: | R | > 0.4 and *P* < 0.001).

Differential Expression Filtering: Differential expression of the candidate DRGs and DRLs was filtered using R software, with thresholds of | Log_2_ (fold change) | > 1 and adjusted *P* < 0.05.

Clinically, a retrospective cohort of 58 GC tissue specimens was collected from the Second Xiangya Hospital. These samples were designated for subsequent RNA extraction and analysis of DRLs expression. All specimens underwent independent histopathological validation to confirm the diagnosis.

### Functional enrichment analysis

Functional enrichment was performed using the Gene Ontology (GO) and Kyoto Encyclopedia of Genes and Genomes (KEGG) pathways analyses with the “ClusterProfiler” and “ggplot2” R packages to interpret the biological effects of the DRGs.

### Construction of a DRLs prognostic model

**Fig. 1:**
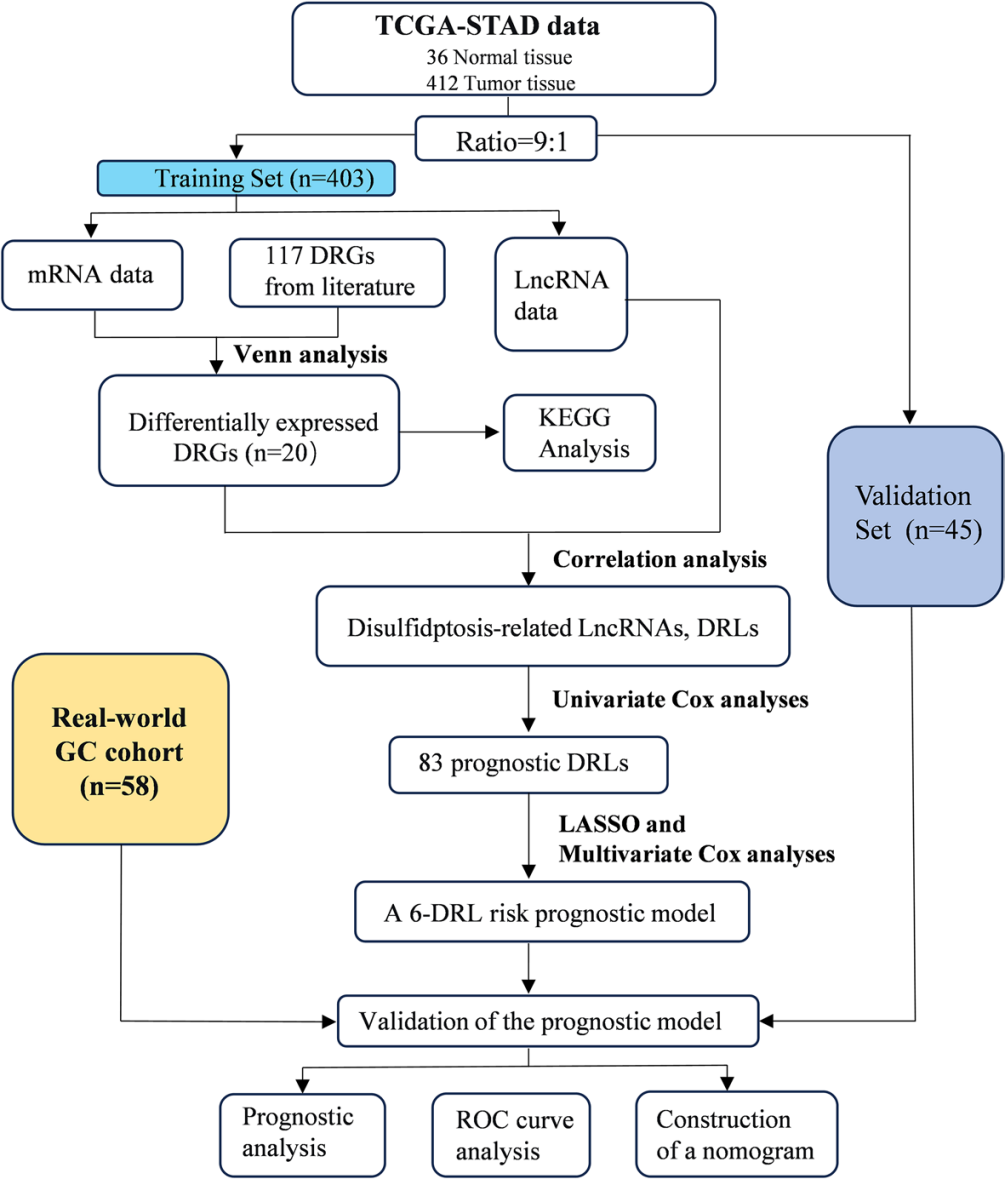
Flowchart for constructing the DRLs prognostic model in GC

Differential expression analysis, univariate Cox proportional hazards regression, and Least Absolute Shrinkage and Selection Operator (LASSO) regression were employed for the preliminary screening of DRLs. Subsequently, a prognostic model was constructed using multivariate Cox regression analysis. Based on the median risk score, patients were stratified into high-risk and low-risk groups. The screening criterion was set at *P* < 0.001. The risk score was calculated using the formula: Risk Score = (Coefficient₁ × Expression level of LncRNA₁) + (Coefficient₂ × Expression level of LncRNA₂) + … + (Coefficientn × Expression level of LncRNAn) (Fig. 1).

### Validation of the prognostic model

The entire cohort of 448 samples from TCGA-STAD was randomly split into a training set (*n* = 403) and a validation set (*n* = 45) at a ratio of approximately 9:1. We evaluated the model’s ability to predict overall survival (OS) via Kaplan-Meier survival analysis, Receiver operating characteristic curve (ROC) curves, and Decision curve analysis (DCA), and developed a nomogram combining DRLs with clinicopathological features to predict 1-, 3-, and 5-year survival probabilities.

### Cell culture

The GC cell lines MKN-45, HGC-27, AGS, and MKN-74, as well as the normal gastric epithelial cell line GES-1, were obtained from the Cell Bank of the Medical Science Research Center at Xiangya Hospital, Central South University. All cells were maintained at 37 °C with 5% CO₂. AGS, HGC-27, MKN-45, and MKN-74 cells were cultured in RPMI-1640 medium (Corning), while GES-1 cells were cultured in DMEM. All media were supplemented with 10% fetal bovine serum (Newzerum, Cat# FBS-500), 100 U/mL penicillin, and 100 µg/mL streptomycin.

###  Quantitative reverse transcription polymerase chain reaction (qRT-PCR)

We extracted total RNA from GC cells and tissues with a Takara RNA kit. Following reverse transcription using Takara’s PrimeScript™ RT kit, real-time PCR was conducted with SYBR Green mix (Takara) on an Applied Biosystems ABI Q7 system (45 cycles). Gene expression was normalized to β-actin. All primer sequences are provided in Table 1.

**Table 1:** Primers used for qRT-PCR analysis

Primer name	Sequence (5'-3')
AL356417.2-F	CACCGAACACCAGTGCAGTAG
AL356417.2-R	GGAGAGAAGGAAGCCACGCAT
β-actin-F	TGGACATCCGCAAAGACCTG
β-actin-R	CCGATCCACACGGAGTACTT

### RNA interference

Three siRNAs targeting AL356417.2 were designed (siDirect online tool) based on its GenBank sequence and synthesized commercially. A non-targeting siRNA (si-NC) served as the control (sequences in Table 2). AGS cells were plated in 6-well plates (1 × 10⁵ cells/well) and transfected at 70% confluency using Lipofectamine 3000. Based on preliminary screening, si-AL356417.2-#1 and si-AL356417.2-#2 were identified as two efficient sequences and were selected for all subsequent functional assays.

**Table 2:** siRNA sequences targeting AL356417.2

siRNA	AntiSense Sequence (5'-3')
si-AL356417.2-#1-	CAAAUGGAAGCUCAUGGUUGA
si-AL356417.2-#2-	CUGACCUCAGGCAGGUGAUCU
si-AL356417.2-#3	GACCUCAGGCAGGUGAUCUGC
si-NC	UUCUCCGAACGUGUCACGUTT

*Stable Cell Line Generation* A lentiviral plasmid for stable AL356417.2 knockdown (sh-AL356417.2) was constructed in the pLKO.1-Puro backbone. Following transduction, cells were selected with 0.5 µg/mL puromycin to establish polyclonal stable lines. We thank Mr. Zhu Zhanwei from Xiangya Hospital for his technical assistance in constructing the lentiviral plasmid.

### Live/dead cell staining and colony formation assay

*Calcein-AM/PI Live/Dead Cell Staining* We employed Calcein-AM and Propidium Iodide (PI) dual-fluorescence staining to evaluate the effect of DRLs on disulfidptosis in GC cells (Kit from Beyotime, Cat# C2015L). Nuclei were labeled with Hoechst 33,342 (Beyotime, Cat# C1029). AGS cells incubated with or without DL-dithiothreitol (DTT, a disulfidptosis inhibitor; MCE, Cat# HY-15917). Following staining, cells were imaged under a fluorescence microscope at 200× magnification.

*Colony Formation Assay* Cells were seeded at a density of 3000 cells per well in 6-well plates and cultured for 7–14 days. The cells were then fixed with 4% paraformaldehyde and stained with 0.1% crystal violet. Colonies containing ≥ 50 cells were counted.

### F-actin staining by phalloidin

The morphology of actin filaments (F-actin) in GC cells was visualized using Actin-Tracker Red-594 (phalloidin conjugate, Beyotime, Cat# C2205S). Briefly, AGS cells were seeded in confocal dishes and cultured to an appropriate density. Cells were fixed with 3.7% formaldehyde at room temperature for 20 min and then permeabilized with PBS containing 0.1% Triton X-100 (washing 3 times, 5 min each). Subsequently, cells were incubated with 200 µL of the diluted Actin-Tracker Red working solution in the dark at room temperature for 60 min. After washing with PBS containing 0.1% Triton X-100 (3 times, 5 min each), the samples were mounted with an antifade mounting medium containing DAPI for nuclear counterstaining. All fluorescent images were acquired using a confocal microscope (Zeiss LSM 900) with a 63× oil-immersion objective.

### Cystine uptake assay

Cystine uptake was quantified using a fluorescence-based assay kit (Elabscience, Cat# E-BC-F066). AGS cells (1 × 10⁵/well in 12-well plates) were trypsinized after overnight culture, washed with PBS, and pelleted. Cell pellets from the si-AL356417.2 group were resuspended in 400 µL of pre-warmed working solution containing the fluorescent cystine analog, while the si-NC control group received PBS. Following a 30-min incubation at 37 °C, cells were centrifuged and lysed with 200 µL ethanol. After a high-speed centrifugation (10,000 × g, 10 min, 37 °C), 50 µL of the supernatant was transferred to a microplate, mixed with 200 µL detection reagent, and incubated in the dark (37 °C, 30 min). Fluorescence (Ex/Em = 485/535 nm) was measured, and the net uptake was calculated by subtracting the control well value from the test well value.

### In vivo xenograft model

Male BALB/c nude mice (4–6 weeks old, *n* = 5 per group) were obtained from Hunan Silek Jingda Experimental Animal Co., Ltd (Changsha, China). To evaluate the in vivo role of DRLs, a subcutaneous xenograft tumor model was established. Mice were randomly assigned to two groups: control (non-targeting shRNA) and experimental (AL356417.2-targeting shRNA). GC cells (1 × 10⁶ in 100 µL PBS) were injected subcutaneously into the right flank. Tumor dimensions were measured with a caliper, and volume was calculated as 0.5 × L × W². A low dose of the disulfidptosis inducer BAY-876 (MCE, Cat# HY-100017) was administered to both control and experimental groups of mice. This compound induces cytoskeletal collapse by inhibiting glucose transport, thereby triggering disulfidptosis [24].

From day 7 post-injection, mice were treated with BAY-876 (1.5 µg/g, subcutaneously, every 3 days). After 4 weeks, tumors were excised, weighed, and processed for fixation (4% PFA) or snap-freezing (-80 °C).

### Immunohistochemical (IHC) and histological analysis

*IHC*: Proliferation was evaluated by Ki67 IHC on 4-µm paraffin sections. After antigen retrieval in citrate buffer (pH 6.0) and blocking, sections were incubated overnight with an anti-Ki67 primary antibody (Zen-bio, Cat# R381101, 1:100) at 4 °C. Signal was detected using an HRP-conjugated secondary antibody and DAB, followed by hematoxylin counterstaining. Ki67-positive nuclei (brownish-yellow) were visualized by light microscopy.

All sections were evaluated independently by two pathologists under double-blind conditions, with disagreements resolved through discussion. Positive staining for Ki-67 protein was localized to the nucleus of tumor cells. Semiquantitative analysis was performed using the immunoreactive score. Five random fields were examined at 400× magnification. Staining intensity was scored as 0 (negative), 1 (weak), 2 (moderate), or 3 (strong). The proportion of positive cells was scored as 0 (0%), 1 (< 25%), 2 (25%–50%), 3 (51%–75%), or 4 (> 75%). The final score was calculated as the product of intensity and proportion scores (range 0–12). Cases were categorized as negative (0), weakly positive (1–4), moderately positive (5–8), or strongly positive (9–12). For statistical analysis, negative and weakly positive cases were defined as the low‑expression group, while moderately and strongly positive cases were defined as the high‑expression group.

*H&E Staining* For general histology, serial sections were deparaffinized, stained with hematoxylin and eosin (H&E) using standard protocols, and examined by light microscopy to assess overall tissue architecture and cytology.

### Statistical analysis

All bioinformatics analyses were performed using R software (version 4.2.0) with Bioconductor packages, including “Limma”, “Survival”, “Survminer”, “ClusterProfiler”, “Glmnet”, and “Dplyr”. For experimental comparisons between two groups, Student’s t-test was applied. All statistical analyses and graphical generation were completed with GraphPad Prism 8 software (GraphPad Software, USA). A two-sided P-value < 0.05 was considered statistically significant.

## Results

### Enrichment analysis of DRLs and model construction in GC

**Fig. 2:**
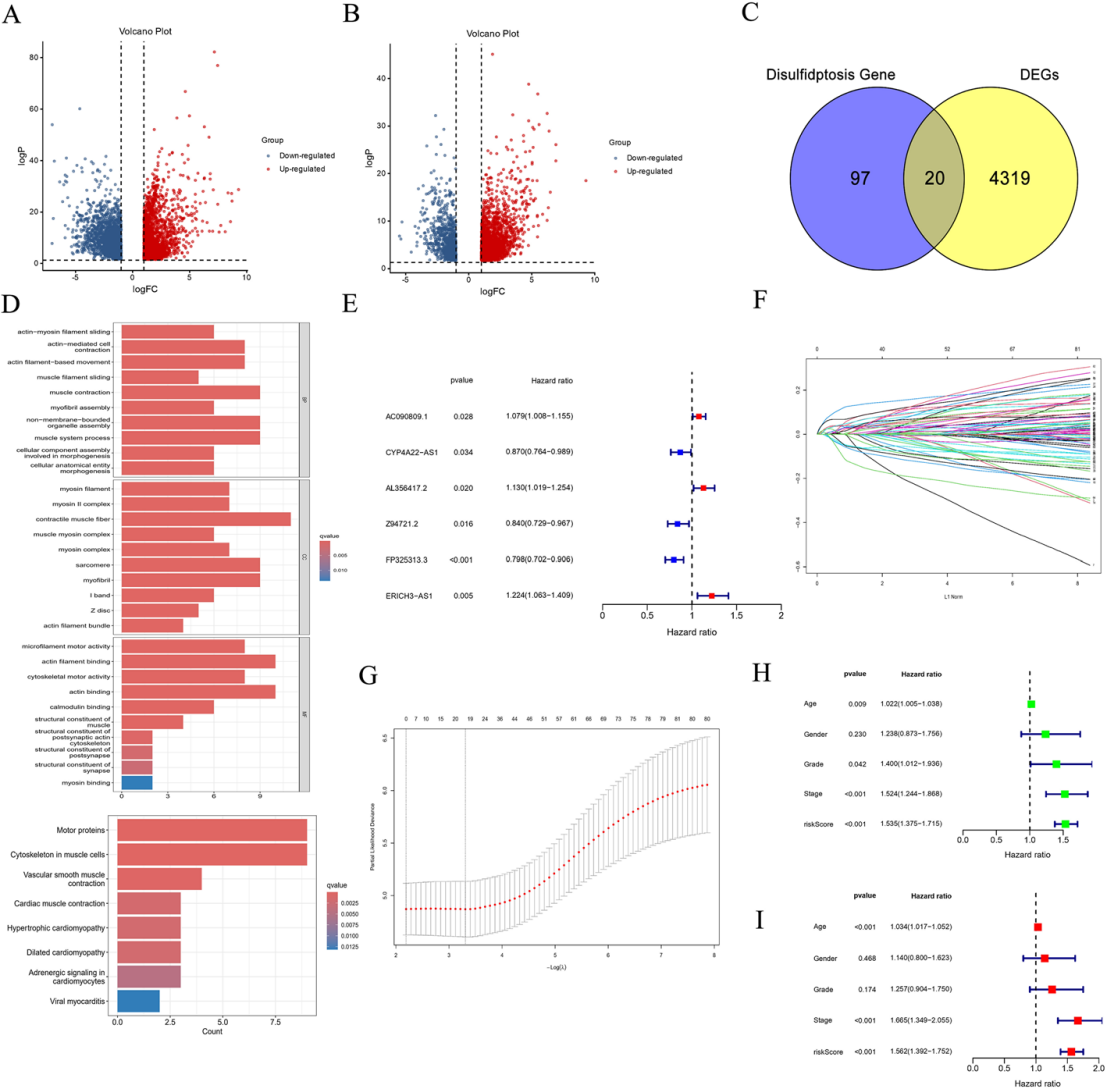
Enrichment analysis of disulfidptosis-related genes and construction of the prognostic model in GC. **A** Volcano plot of differentially expressed mRNAs in the TCGA-STAD dataset; **B** Volcano plot of differentially expressed LncRNAs in the TCGA-STAD dataset; **C** Venn diagram identifying 20 differentially expressed DRGs by intersecting known 117 DRGs with differentially expressed mRNAs. **D** GO enrichment analysis of differentially expressed DRGs, showing the top significantly enriched terms in Biological Process (BP), Molecular Function (MF), Cellular Component (CC) categories and Kyoto Encyclopedia of Genes and Genomes (KEGG) pathway enrichment analysis for the differentially expressed DRGs. **E** 6 DRLs preliminarily identified by univariate Cox regression analysis. **F** & **G** Further screening of prognosis-related DRLs using the LASSO regression analysis. **H** & **I** Univariate (up) and multivariate (down) Cox regression analyses evaluating independent prognostic factors for GC

We extracted RNA sequencing data from TCGA-STAD and intersected differentially expressed mRNAs with a published list of 117 DRGs via Venn analysis. This yielded 20 differentially expressed DRGs in GC patients (FDR < 0.05, |log2FC| > 1). In order to further investigate the biological pathways of these DRGs, we performed GO and KEGG enrichment analysis. GO enrichment analysis was categorized into three main domains: biological process (BP), cellular component (CC), and molecular function (MF). The top 10 significantly enriched terms in each category were identified based on qvalue. In the BP category, DRGs were predominantly enriched in muscle contraction and cytoskeleton organization processes, including actin-myosin filament sliding, muscle filament sliding, myofibril assembly, and others. In the CC category, DRGs were significantly enriched in myofibril and sarcomere-related components, such as contractile muscle fiber, sarcomere, and myofibril. In the MF category, DRGs were primarily associated with structural and motor functions, such as microfilament motor activity, actin filament binding, cytoskeletal motor activity, and actin binding. KEGG pathway analysis revealed significant enrichment in pathways related to motor proteins and cytoskeleton organization in muscle cells (Fig. 2D).

LncRNAs correlated with the 20 DRGs were screened via Pearson analysis, from which 83 DRLs associated with OS were initially selected by univariate Cox regression. Further refinement using LASSO regression (Fig. 2E, F and G) and multivariate Cox analysis identified 6 (*AC090809.1*,* CYP4A22-AS1*,* AL356417.2*,* Z94721.2*,* FP325313.3*,* ERICH3-AS1*) independent prognostic DRLs. A prognostic risk score was computed for each patient by multiplying the expression level of each of these 6 LncRNAs by its respective multivariate Cox regression coefficient and summing the products. Univariate Cox regression analysis showed that Age, Grade, Stage and risk score were significantly associated with OS (*P* < 0.05, Fig. 2H), and Multivariate Cox regression show that Age, Stage and risk score could act as independent prognostic factor (*P* < 0.05, Fig. 2I). Thereby establishing a model based on 6 independent prognostic predictors.

### Validation of the DRLs prognostic signature

**Fig. 3:**
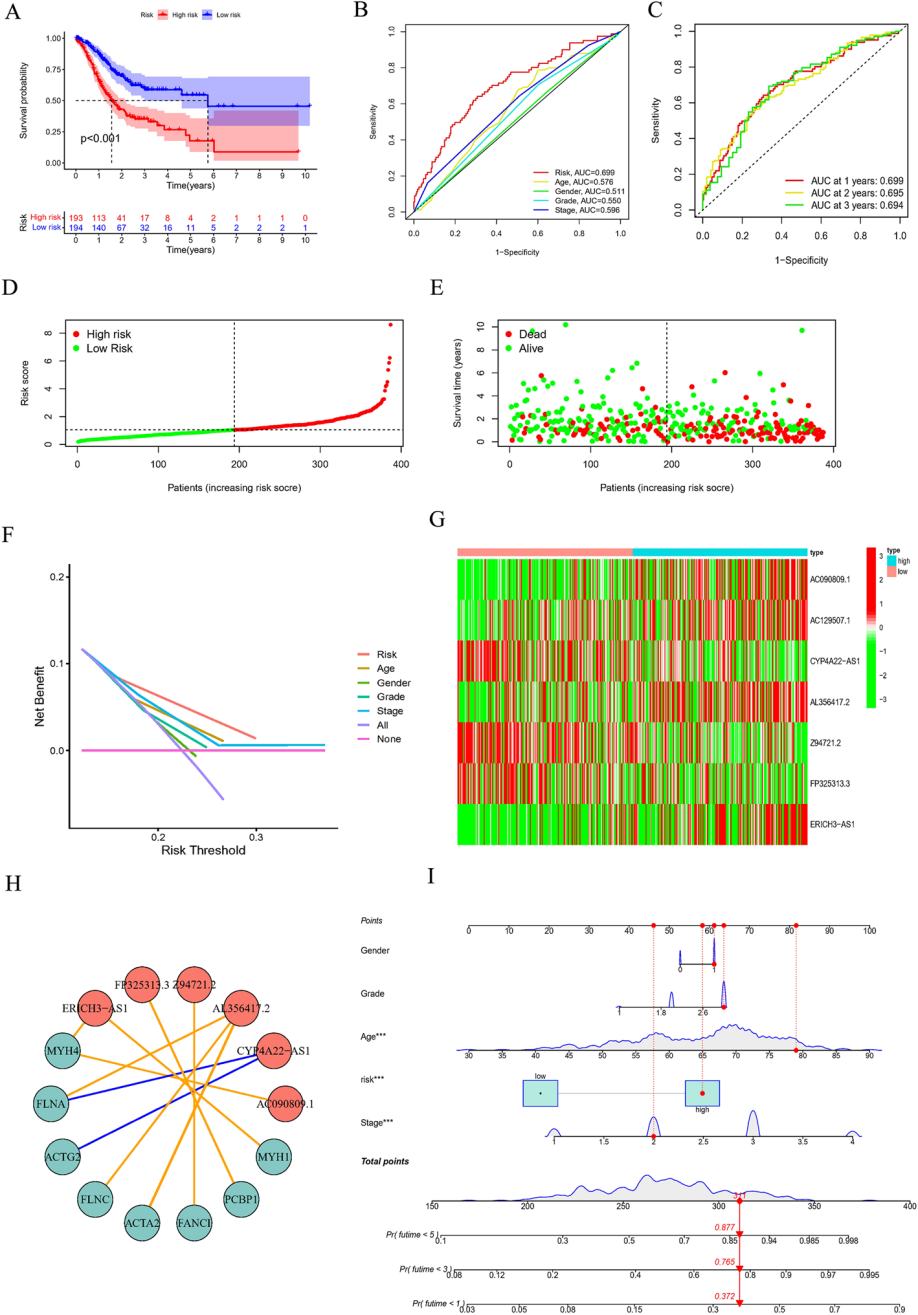
Validation of the DRLs Prognostic Signature. **A** Survival curves result; **B** & **C** Predictive accuracy (AUC) of clinical characteristics and the independent risk score for OS; **D** & **E** Risk score distribution and survival status of stomach cancer patients; **F** The DCA plot; **G** Expression profile heatmap of the 6 DRLs signature. H The Network diagram of LncRNA-mRNA relationships (I)Construction of a hybrid nomogram based on both prognostic DRLs and prognostic-related clinicopathological factors

Stratification by the median risk score effectively distinguished patients with significantly different outcomes, as evidenced by Kaplan-Meier curves (Fig. 3A) and a clear correlation between higher risk scores and increased mortality (Fig. 3D and E). The model showed consistent predictive power, with an AUC of 0.699 for OS (Fig. 3B) and promising 1-, 2-, and 3-year AUCs of 0.699, 0.695, and 0.694, respectively (Fig. 3C). Its clinical utility was supported by DCA (Fig. 3F). The correlations among DRGs and DRLs are shown in Fig. 3H, and a comparative nomogram highlights the model’s additive value (Fig. 3I).

**Fig. 4:**
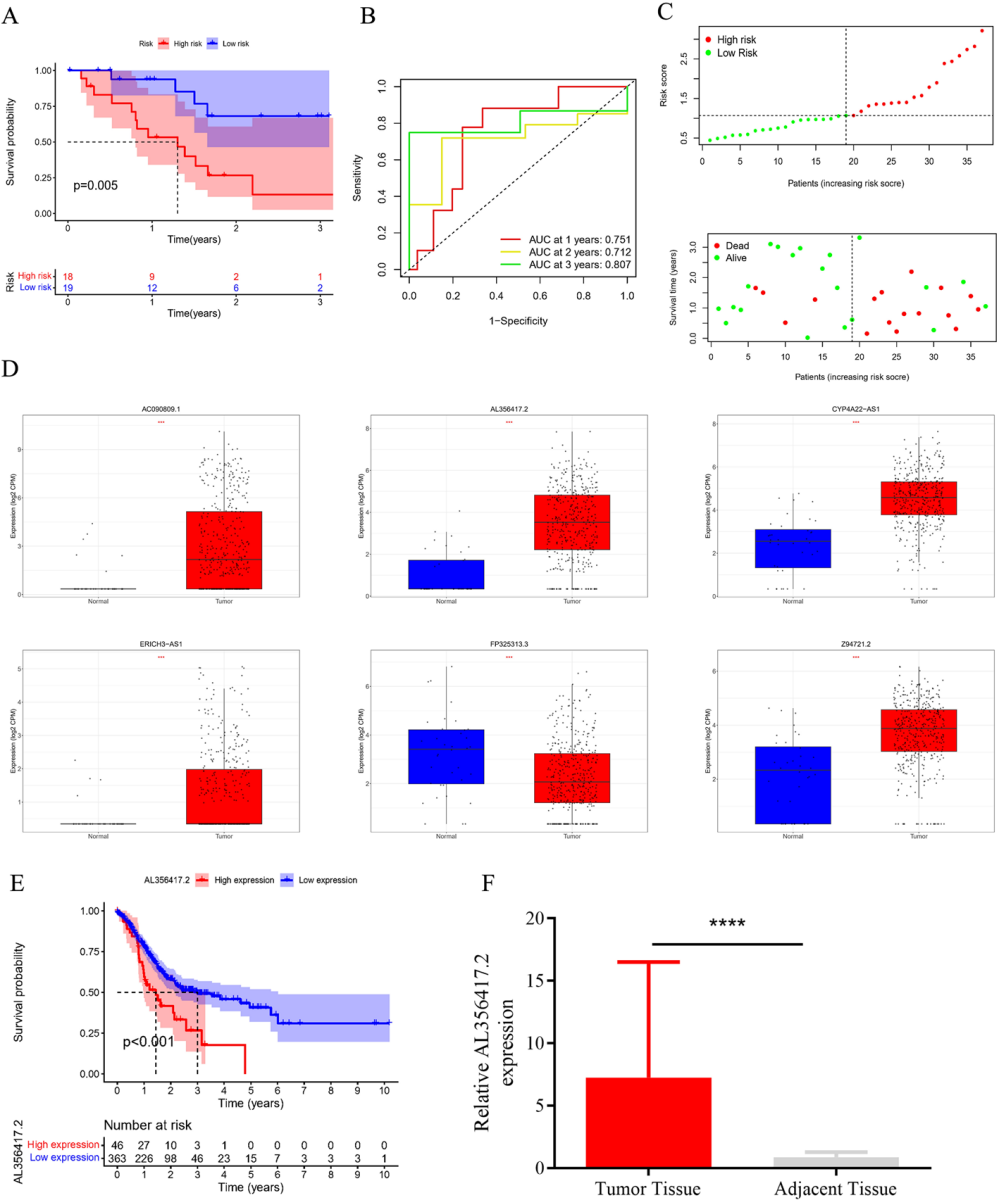
External Validation of the 6-DRL Prognostic Signature. **A** Analysis of the associations between differential expression of the six DRLs and survival outcomes in GC; **B** Predictive accuracy (AUC) of the independent risk score for OS; **C** Risk score distribution and survival status of stomach cancer patients; **D** Comparative analysis of the six DRGs expression: Box plots of their levels in GC tumors versus matched adjacent tissues. **E** Kaplan-Meier survival analysis comparing overall survival between TCGA-STAD patients with high versus low expression of AL356417.2. **F** Validation of AL356417.2 expression in 58 paired clinical GC specimens by qRT-PCR

Subsequently, we evaluated the performance of the 6-DRL prognostic signature in an independent validation cohort. As shown in Fig. 4A, patients in the high-risk group exhibited a significantly shorter OS compared to those in the low-risk group (*P* < 0.05). The time-dependent receiver ROC analysis demonstrated that the signature achieved area under the curve (AUC) values of 0.751, 0.712, and 0.807 for predicting 1-, 2-, and 3-year survival, respectively (Fig. 4B). Furthermore, a positive correlation was observed between the increasing risk score and patient mortality (Fig. 4C). Collectively, these results validate the DRLs signature as a reliable and independent prognostic tool for GC.

To elucidate the functional relevance of these six DRLs in GC, we first analysed their expression profiles in the TCGA-STAD cohort. The expression levels of AC090809.1, CYP4A22-AS1, AL356417.2, Z94721.2, and ERICH3-AS1 were significantly upregulated in tumour tissues compared with adjacent non-tumour tissues, with fold change (FC) values of 4.69, 2.22, 3.78, 1.42, and 2.46, respectively (Fig. 4D). In contrast, FP325313.3 showed a lower expression in tumours (FC: 1.2). Based on preliminary qPCR experiments and literature review, we focused subsequent investigations on AL356417.2. Kaplan-Meier analysis revealed that patients with high AL356417.2 expression had a markedly worse OS outcome (Fig. 4E). Consistent with the bioinformatics findings, the expression level of AL356417.2 was significantly elevated in tumour samples from our clinical validation cohort compared to matched adjacent tissues, with an average increase of 8.18-fold (*P* < 0.05, Fig. 4F).

### Functional role of AL356417.2 in GC

**Fig. 5:**
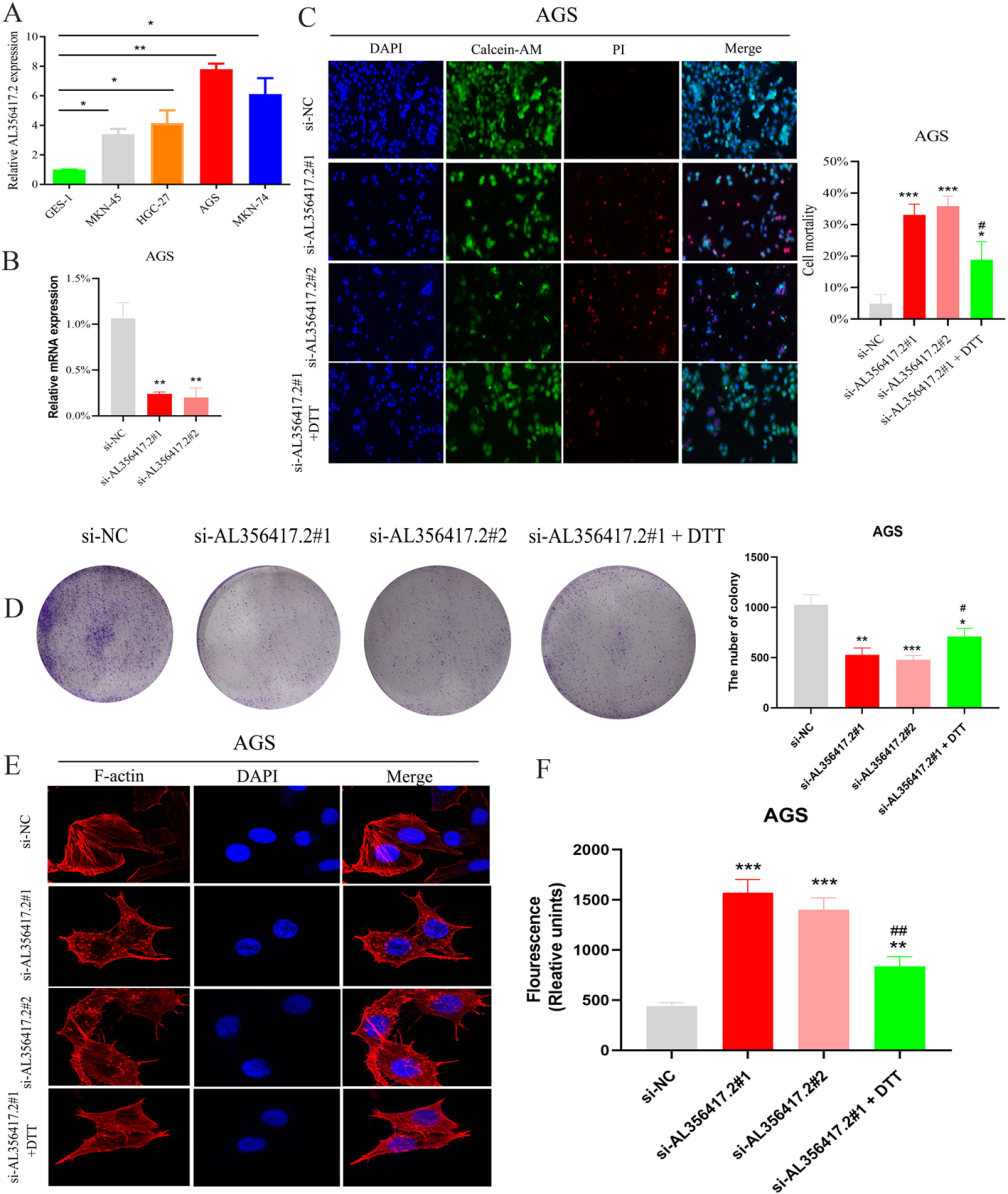
Functional validation of AL356417.2 in GC cells in vitro. **A** Expression levels of AL356417.2 in the normal gastric epithelial cell line GES-1 and GC cell lines (MKN-45, HGC-27, AGS, MKN-74) as detected by qRT-PCR. **B** Knockdown efficiency of AL356417.2 was validated by qPCR following transfection with two specific siRNAs. **C** Cell viability assessed by Calcein-AM (green, live cells) / Propidium Iodide (PI, red, dead cells) staining following AL356417.2 knockdown. **D** The effect of AL356417.2 knockdown on the growth capacity of GC cells was assessed using a colony formation assay. **E** Morphology of F-actin stained by phalloidin in GC cells under a confocal microscope (63× oil objective). F Effect of AL356417.2 knockdown on cystine uptake capacity in GC cells. Compared with the si-NC group, *: $\:P<0.05$; **: $\:P<0.01$; ***: $\:P<0.001$. Compared with the si-AL356417.2 group, #: $\:P<0.05$; ##: $\:P<0.01$

In vitro, AL356417.2 expression was significantly elevated in multiple GC cell lines compared to the normal gastric epithelial cell line GES-1, with the highest level observed in AGS cells (Fig. 5A).

Knockdown of AL356417.2 with two independent siRNAs (si-AL356417.2-#1 and #2) significantly reduced cell viability compared to the si-NC control, as assessed by Calcein-AM/PI live/dead staining (*P* < 0.05, Fig. 5C). Phalloidin staining for F-actin revealed dramatic cytoskeletal remodeling upon AL356417.2 knockdown: while control cells displayed well-organized cortical actin filaments, knockdown cells exhibited characteristic F-actin polymerization and detachment from the plasma membrane (Fig. 5E). Concurrently, cystine uptake assays demonstrated a significant increase in cystine uptake in both knockdown groups compared to the control (*P* < 0.05, Fig. 5F). To further validate the effect of AL356417.2 on disulfidptosis in GC cells, we performed rescue experiments using DTT, a disulfidptosis inhibitor. Live/dead cell staining and colony formation assays revealed that DTT treatment significantly restored cell viability in the si-AL356417.2 group. Meanwhile, DTT was observed to markedly reverse F-actin polymerization, restoring the orderly arrangement of the cytoskeleton. Additionally, cystine uptake was also significantly reversed (*P* < 0.05, Fig. 4C, D and E).

Collectively, these results indicate that silencing AL356417.2 induces a disulfidptosis-like phenotype in AGS cells.

### AL356417.2 knockdown suppresses tumour growth in vivo

**Fig. 6:**
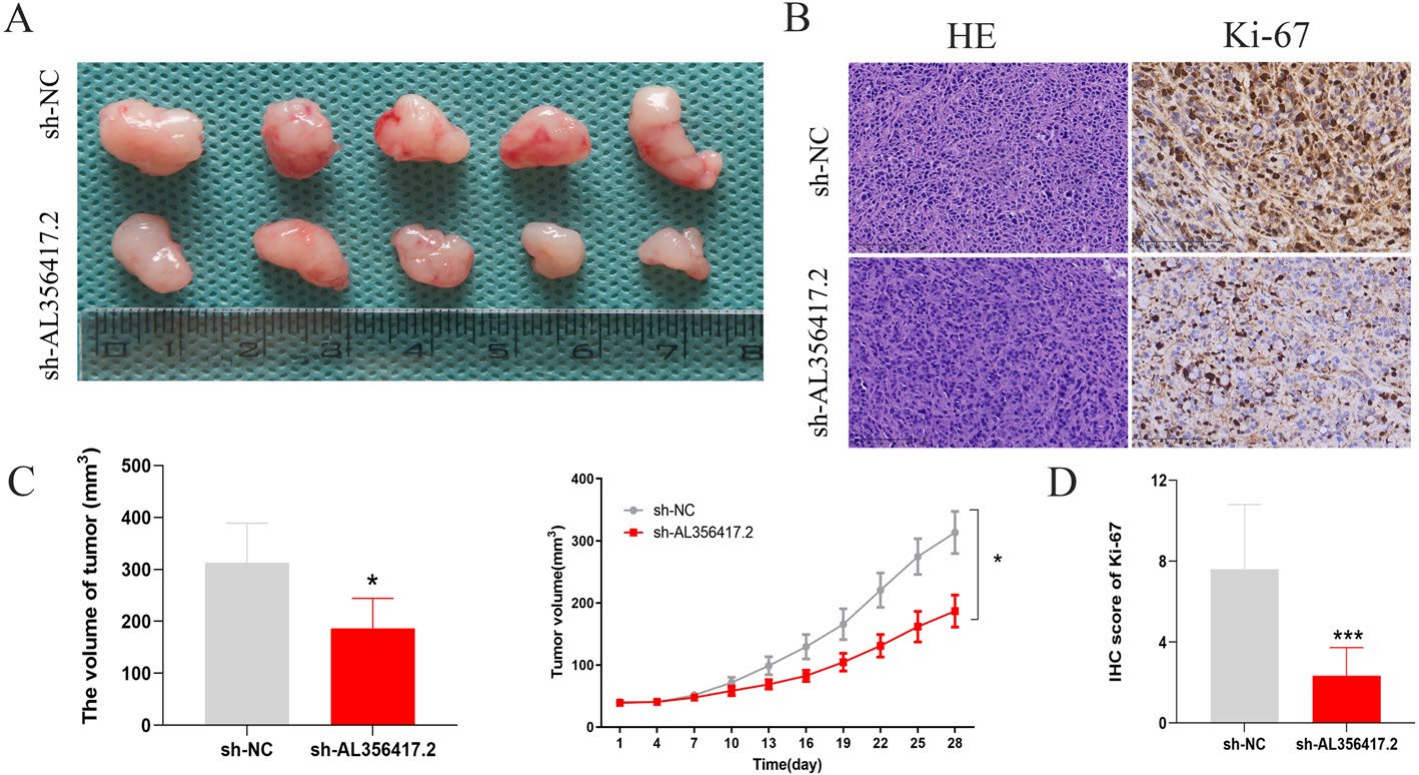
AL356417.2 knockdown suppresses tumour growth in vivo. **A** & **C** Representative gross images of dissected tumors from the subcutaneous xenograft mouse model. **B** & **D** Representative images of H&E staining and immunohistochemical (IHC) analysis for Ki-67 expression in tumor tissues. Compared with the sh-NC group, *: $\:P<0.05$; ***: $\:P<0.001$

To validate the pro-tumorigenic role of AL356417.2 observed in vitro, we established a xenograft model with stably silenced AL356417.2. Accordingly, knockdown of AL356417.2 significantly inhibited subcutaneous tumour growth, as evidenced by reduced tumour volume and the tumour growth curve (*P* < 0.05, Fig. 6A and C) and decreased expression of the proliferation marker Ki-67 (*P* < 0.05, Fig. 6B and D). These in vivo findings reinforce the conclusion that AL356417.2 promotes GC progression by mitigating disulfidptosis.

## Discussion

Disulfidptosis is a novel cell death pathway characterized by disulfide stress due to cystine overload during glucose deprivation. This stress promotes aberrant disulfide bond formation in actin, causing cytoskeletal collapse and rapid cell death [12, 13]. Notably, tumour cells exhibit a distinct susceptibility to disulfidptosis due to their high metabolic activity and heavy reliance on cystine uptake, rendering this novel cell death pathway a promising strategy for cancer therapy [25, 26]. For instance, Chen et al. reported that QSOX2-mediated disulfidptosis enhances tumour stemness in esophageal squamous cell carcinoma (ESCC) by activating the mTOR signalling pathway [27]. Furthermore, prognostic models constructed based on disulfidptosis-related signatures have demonstrated significant predictive value in various cancers, including prostate cancer and melanoma [28–30] .

Through systematic analysis, we defined a 6-DRL prognostic signature for GC, comprising *AC090809.1*,* CYP4A22-AS1*,* AL356417.2*,* Z94721.2*,* FP325313.3*,* ERICH3-AS1.* Consistent with their emerging roles in tumor biology, key constituents of our signature, such as CYP4A22-AS1 and AL356417.2, have been associated with prognosis in other cancers including lung and breast cancer [31–33]. Beyond prognostic value, specific functional roles in GC are emerging. CYP4A22-AS1 has been implicated in hypoxic/angiogenic processes and correlates with immune checkpoint expression in GC [34]. Similarly, ERICH3-AS1 is reported to contribute to gastric tumorigenesis potentially by modulating critical signaling pathways (e.g., ERBB, MAPK, MTOR, P53, and Wnt) that regulate cell cycle and apoptosis, and its high expression is linked to poorer patient outcomes [35, 36]. However, the precise functional mechanisms and biological roles of these molecules in tumorigenesis remain largely unexplored.

We developed and validated a prognostic risk-score model by integrating the expression of these 6 DRLs with standard clinicopathological features. This model effectively stratified GC patients, with high-risk scores being significantly correlated with adverse prognosis, underscoring its clinical relevance. Strikingly, the DRLs were enriched in cytoskeleton-associated processes like " Actin binding, cytoskeleton and Motor proteins " which is consistent with the defining cytological feature of disulfidptosis and reinforces its probable significance in gastric carcinogenesis [27, 37]. To further investigate the potential functions of the identified DRLs in GC, we analyzed their expression differences between tumor and adjacent normal tissues in the TCGA-GC cohort. In preliminary functional screening, knockdown of AL356417.2 induced more pronounced cellular phenotypic changes, including reduced cell viability and cytoskeletal remodeling, suggesting its prominent biological effects. In addition, compared with other candidate molecules (e.g., AC090809.1), AL356417.2 exhibited more stable and efficient interference in cellular models, making it more suitable for subsequent functional studies. Moreover, AL356417.2 has been suggested as a potential biomarker associated with prognosis and immunotherapy efficacy in GC [38]. This finding was further validated in our independent, retrospectively collected cohort of 58 surgical GC specimens, reinforcing its potential role in GC progression. Therefore, we selected AL356417.2 for subsequent studies.

In vitro, AL356417.2 expression was significantly elevated in multiple GC cell lines relative to the normal gastric mucosal epithelial cell line GES-1. Notably, knockdown of AL356417.2 induced distinct morphological alterations in GC cells, characterized by F-actin depolymerization and, in some cells, detachment of F-actin from the plasma membrane. This phenotype aligns with the characteristic disruption of the actin network caused by disulfide stress during disulfidptosis [39]. Furthermore, the cystine uptake assay demonstrated that AL356417.2 knockdown significantly enhanced cystine uptake capacity alongside a marked increase in cell death. To further validate the role of AL356417.2 in disulfidptosis in GC cells, we performed rescue experiments using the disulfidptosis inhibitor DTT. The results showed that DTT treatment significantly restored cell viability, which had been impaired by AL356417.2 knockdown, and reversed F-actin polymerization, leading to the restoration of orderly cytoskeletal arrangement in the si-AL356417.2 group. In addition, cysteine uptake was also significantly rescued. Collectively, these findings suggest that AL356417.2 may promote GC cell survival by conferring resistance to disulfidptosis, potentially through modulating cystine metabolism and cytoskeletal stability.

To summarize, we developed and validated a DRLs prognostic signature for GC. We identified and functionally characterized AL356417.2 as a crucial mediator that confers resistance to disulfidptosis, likely via modulating the cystine-actin axis, and its clinical relevance was established in multiple cohorts. We acknowledge that the clinical translation of this prognostic model requires further external validation in multicenter, prospective cohorts to better assess its generalizability and clinical applicability. While our work offers new insights into GC prognosis and biology, future studies are needed to decipher the exact mechanism of AL356417.2 and to explore the roles of the other six DRLs in GC.

## Supplementary Information

Below is the link to the electronic supplementary material.

Supplementary Material 1.

## Data Availability

The datasets analyzed during the current study are publicly available in the Genomic Data Commons (GDC) Data Portal of The Cancer Genome Atlas (TCGA). The data can be accessed via the following link: https://portal.gdc.cancer.gov/projects/TCGA-STAD. The specific data used in this study were filtered by selecting the following criteria: Data Category​ = “Transcriptome Profiling”, Data Type​ = “Gene Expression Quantification”, and Workflow Type​ = “HTSeq - Counts”. Specifically, after downloading the raw data from TCGA-STAD, we performed differential expression analysis using DESeq2 and subsequently normalized the count matrix using edgeR. The final normalized expression matrix for the 448 GC samples is provided as Supplementary File “Supplementary normalized\_counts\_448 samples.csv”. All original data generated during this research are available from the corresponding author upon reasonable request.
